# Editorial: Advances and controversies in skull base tumors: implication for diagnosis, treatment and management

**DOI:** 10.3389/fonc.2024.1377868

**Published:** 2024-03-25

**Authors:** Diego Mazzatenta, Arianna Rustici, Carlo Serra

**Affiliations:** ^1^ Department of Biomedical and Neuromotor Sciences (DIBINEM), University of Bologna, Bologna, Italy; ^2^ IRCCS Istituto delle Scienze Neurologiche di Bologna, Programma Neurochirurgia Ipofisi – Pituitary Unit, Bologna, Italy; ^3^ IRCCS Istituto delle Scienze Neurologiche di Bologna, UOSI Neuroradiologia – Ospedale Maggiore, Bologna, Italy; ^4^ Department of Neurosurgery, Clinical Neuroscience Center, University Hospital Zurich, University of Zurich, Zurich, Switzerland

**Keywords:** skull base, advances and innovations in skull base, advanced therapies, endoscopic surgery, imaging biomarker, tumor, innovative surgical approaches

The skull base has long been regarded as one of the Pillars of Hercules of neurological pathologies. This is because, in addition to being a difficult surgical site, it is situated on the boundary between the nasal, paranasal, neck regions and the central nervous system. Moreover, it presents a number of vascular-nervous structures that have historically caused diagnostic challenges. In this anatomic region a huge number of pathological processes, including neoplasms ranging in nature from benign to malignant originating from each of the border regions that make up the base of the skull, as well as distant heteroplastic processes can be found.

This Research Topic focused on the Advances and Controversies in Skull Base Tumors: Implication for Diagnosis, Treatment and Management. Out of the 18 articles in this Research Topic, there are 8 original articles, 4 reviews, 2 systematic reviews, 3 case reports, and one opinion-based article.

## Common skull base tumor pathologies and biomarkers

Although the most common pathologies are now well recognized, some of them should not be undervalued as they can resemble “common” skull base tumors. This is demonstrated in the publication by Bove et al. Furthermore, Ghezzi et al. showed in their case report that tumors that infrequently metastasize to the skull base region can also involve it, and thus a multidisciplinary team that takes into account both preoperative imaging and postoperative histopathological findings is required in order to ensure better management for each patient.

Even though a preoperative diagnosis of certainty has not yet been questioned, Wijethilake et al. work offers a thorough summary of the noteworthy advancements in the fields of molecular biology and biochemical markers for skull base tumors. They explain the relationships between the histotypes of skull base tumors and growth-related parameters, such as grade, survival, growth/progression, recurrence, and treatment outcomes.

In another article published in this Research Topic, Righi et al. correlated a new protein mutation with clinicopathological parameters and survival outcomes in a group of poorly differentiated chordomas, which also included possible treatment suggestions.

However, the increasing need to guarantee an excellent outcome for patients has led to the identification of several preoperative biomarkers, some of which involve relatively simple preoperative execution. Takahara et al.’s retrospective series of Schwannomas serves as an example of this. Their study demonstrated a relationship between the preoperative neutrophil/lymphocyte ratio (NLR) and postoperative recurrence as well as treatment-free survival rate.

## Current status of skull base surgery for major tumor pathologies

On the other hand, skull base surgery has seen a succession of historical phases, each differentially characterized in terms of objectives, techniques and problems, each inevitably influenced also in a broader perspective by the evolution of society and its needs and expectations, and the neurosurgeon is daily confronted with this challenge.

Where does skull base surgery stand today? Zhang et al. in an interesting review of scientometry on the state of research on meningiomas, indicate what Wang et al., Na et al. and Baussart et al. confirm for us in their clinical studies on the current, we would say consolidated, status of skull base neurosurgery with regard to pathologies of greater epidemiological significance in the neurosurgical field: meningiomas, craniopharyngiomas, pituitary adenomas and schwannomas.

## Incorporating patient-specific factors into surgical planning

However, while the clinical studies comfort us and indicate that the possibility of more than satisfactory results can now be considered established in terms of tumor removal and surgical morbidity, Zhang et al. warns us of a change taking place, namely, of the need increasingly felt by both clinicians and, as we shall later see, patients, to try to understand pathology not only in terms of nosologic macrocategories but also in terms of the single, unique and, (exaggerating somewhat), unrepeatable pathology in the individual, and this yes, unrepeatable, patient.

In the case of the scientometry trend, this is expressed in the increasing focus of scientific literature and thus scientific research on more and more detailed molecular characterizations, which is nothing different than a reflection on the progress of medicine toward more and more personalized medicine. In other words, toward a possible future where artificial intelligence will help make sense of the huge amount of data that we are increasingly gathering about patients and patients’ pathology.

The trend, however, can be seen in the clinical setting as well: this is now so true, and to such an extent, that neurosurgical practice can and must also be able to respond to n new needs of specific patient populations that are no longer the same as that with which the “noble fathers” of skull base neurosurgery in the 1970s and 1980s were confronted in their time. These are, at least in the Western world, patients who are on average increasingly frail and increasingly concerned with what is called “quality of life”. The Tübingen group, on the strength of its experience and undisputed expertise in the microneurosurgery of schwannomas of the VII cranial nerve indicates to us on the one hand that advanced age is not a limitation even in surgeries once considered “high-risk”, as vestibular schwannoma surgery might have been (Wang et al.), on the other hand how the focus should now be placed not only on the extent of resection but also if not especially on other indirect health parameters, such as mental health (Machetanz et al.) and obviously, as also suggested by Di Perna et al., on functional outcome. This is also seen, however, in the need to propose solutions, be they purely neurosurgical or interdisciplinary in nature, that can meet the needs of the individual case.

## Optimizing surgical outcomes and safety, including endoscopy

Furthermore, current research efforts are also aimed at the optimization of the surgical results and safety during vestibular schwannoma resection. Vychopen et al., by the means of a systematic review, attempt to add evidence to the longstanding discussion of the patient positioning during these procedures, reporting that both the semisitting and the lateral position are safe, with a possible superiority of the first in the functional outcome of facial nerve. Concurrently, Yang et al. report their experience in the endoscopic assistance during microsurgical procedures, with good results. They add further corroborating evidence to this technique, which enhances the surgical view potentially improving the safety of the procedure and, ultimately, of the patient himself.

The endoscope itself could be considered as a cornerstone instrument in skull base neurosurgery. Its widespread diffusion, affordability and flexibility made it irreplaceable. In addition to its well – known use, for example for the management of sellar, parasellar and anterior skull base pathologies or for the exploration of the cerebello – pontine angle (as previously reported by Authors), surgeons and researchers are now pushing the boundaries further. Bai et al. describe in detail for the first time the endoscopic far lateral supracerebellar infratentorial approach, which was successfully used *in vivo* for the resection of a posterior clinoid meningioma. The same authors complete their report with a systematic review focused on the treatment of this rare pathology. Furthermore, Yan et al. analyze their case series of endoport – assisted neuroendoscopic resection of lateral ventricle tumors. The endoscope will clearly retain its paramount role in skull base surgery, as the enhancement of the surgical view, by the means of advancing the “surgeon’s eye” deep in the surgical field, is crucial to navigate and operate in anatomical areas full of delicate vascular and neural structures.

It should also be clear that surgery does not stand alone as the only treatment of skull base pathologies. Radiation therapy, by the means of conventional external beam radiotherapy or particle therapy, as extensively outlined by Iannalfi et al. is complementary to surgery, and should also always discussed in a multidisciplinary setting, with close collaboration between experts different as clearly stated by Carsuzaa et al.

A multidisciplinary management setting is also a key to provide the best treatment for olfactory neuroblastomas. This concept was clearly outlined by Tosoni et al., who extensively review and report the current knowledge and clinical practice concerning this uncommon clinical entity. While surgical resection with established skull base techniques and radiation therapy remains the mainstays of the treatment, the Authors analyze the possible role of chemotherapy protocols, which could be of the utmost importance especially in cases of advanced disease, while advocating also for forward - looking experimentation of targeted therapies basing on molecular profiling.

With the help of this Research Topic and the many articles it includes, one can obtain a comprehensive 360-degree view of the current debates surrounding the diagnosis and management of skull base pathologies. While the current guidelines for treating diseases of the skull base have been reaffirmed in this Research Topic, new developments in biomolecular, diagnostic, and surgical technologies have also been included to enhance patient outcomes. Because of their patient-centered approaches, the articles in this Research Topic provide a foundation for identifying the key areas of future research and development.

## Author contributions

DM: Conceptualization, Data curation, Formal analysis, Investigation, Methodology, Project administration, Software, Supervision, Validation, Visualization, Writing – original draft, Writing – review & editing, Funding acquisition, Resources. AR: Conceptualization, Data curation, Formal analysis, Investigation, Methodology, Project administration, Software, Supervision, Validation, Visualization, Writing – original draft, Writing – review & editing. CS: Conceptualization, Data curation, Formal analysis, Investigation, Methodology, Project administration, Software, Supervision, Validation, Visualization, Writing – original draft, Writing – review & editing, Funding acquisition, Resources.

